# Outcome Evaluation Affects Facial Trustworthiness: An Event-Related Potential Study

**DOI:** 10.3389/fnhum.2020.514142

**Published:** 2020-11-10

**Authors:** Haizhou Leng, Ying Liu, Qian Li, Qi Wu, Dong Li, Zhongqing Jiang

**Affiliations:** ^1^Element Education Department, Hebei Normal University, Shijiazhuang, China; ^2^School of Psychology, Liaoning Normal University, Dalian, China; ^3^Xingtai Special Education School, Xingtai, China

**Keywords:** event-related potential, facial trustworthiness, feedback-related negativity, outcome evaluation, trust game

## Abstract

Facial trustworthiness and feedback information of trustees can influence trustors’ investment behavior in trust games. This study investigated the temporal features of outcome evaluation (evaluation of feedback) and how they influence the processing of facial trustworthiness. A total of 25 college students participated in a decision-making task in which feedback was presented prior to a face stimulus. The decision of participants to continue investing was evaluated. We observed that trustors were more inclined to keep investing in trustworthy trustees or those appearing after positive feedback (gains). Event-related potential (ERP) results revealed that in the face presentation stage, trustworthy faces with losses induced more negative feedback-related negativity (FRN) than did trustworthy faces with gains and untrustworthy faces with losses. Further, faces that did not meet expectations induced more negative FRN. Trustworthy faces with gains induced more positive late positive component (LPC) than did trustworthy faces with losses and generated more motivated attention. Bottom–up and top–down processes were integrated for facial trustworthiness perception at different stages. In sum, top–down processing exerted a greater impact during the early stage of facial trustworthiness perception, both top–down and bottom–up processing were involved in the medium term, and bottom–up processing exerted a greater impact in the later stage.

## Introduction

Facial trustworthiness perception is a face-based trait inference process that refers to the evaluation of others’ trustworthiness based on their faces. The perception of facial trustworthiness is a fast process; indeed, individuals can judge the trustworthiness of a face when it appears for 33 ms (Todorov et al., 2009). Judging the trustworthiness of a stranger’s face is a spontaneous process (Klapper et al., 2016) that does not require effort of will (Bonnefon et al., 2013). This perceptual feature of facial trustworthiness is of great significance for the survival and development of humans.

Facial trustworthiness perception provides key information about whether someone should be approached or avoided, which can be influenced by the emotional state of the perceiver and target (Oosterhof and Todorov, 2008; Meconi et al., 2014). A trustworthy face can be considered to convey negative information to an extent; i.e., perception of trustworthiness is positively correlated with judgments of happiness and negatively correlated with the perception of anger from emotionally neutral faces (Oosterhof and Todorov, 2008). Event-related potential (ERP) studies have revealed that untrustworthy faces elicited greater electrical activity in several stages of face processing than did trustworthy faces, as demonstrated in P100, N170, early posterior negativity, feedback negativity (FN), and late positive component (LPC)/late positive potential (LPP; Yang et al., 2011; Marzi et al., 2012; Li et al., 2017; Lischke et al., 2018). Li et al. (2017) found that untrustworthy faces induced more negative FN than trustworthy faces in the late phase of the game and that the anterior cue-elicited FN reflects the reputation appraisal and tracks the reputation learning process in social interactions. People are more inclined to stay away from untrustworthy face individuals, and pay more attention to untrustworthy faces, which induced more positive LPC (Schupp et al., 2000, 2004a,b; Langeslag et al., 2007; Yang et al., 2011). However, studies have also reported that trustworthy faces aroused more positive ERP than did untrustworthy faces, such as an enhanced positivity at approximately 150 ms at frontal sites (Marzi et al., 2012) and during the time window of 200–400 ms over the frontal lobe (Rudoy and Paller, 2009).

Berg et al. (1995) designed a trust game in which the trustor is first given $10 and then must decide how much to “invest” in the trustee. When the amount of investment triples, it is at the discretion of the trustee to decide how much to repay. Facial trustworthiness plays a critical role in the social perception of faces and directly determines the establishment and development of interpersonal trust (Winston et al., 2002; Oosterhof and Todorov, 2008; Van’t Wout and Sanfey, 2008). Individuals prefer to lend money to others who look trustworthy Duarte et al., 2012; Jenq et al., 2015). In trust games, trustors invest more money in trustees with trustworthy faces than in those with untrustworthy faces (Van’t Wout and Sanfey, 2008; Chang et al., 2010; Rezlescu et al., 2012; Tingley, 2014; Ewing et al., 2015; Bailey et al., 2016).

The influence of facial trustworthiness on decision making in trust games may be reduced when participants learn about their partners’ past related behaviors (Chang et al., 2010; Rezlescu et al., 2012). Outcomes (trustees’ feedback) will influence trustors’ investment behavior. With an increase in communication, trustors pay more attention to trustees’ behavior, the influence of the face gradually decreases, and trustors invest more money in partners who repay more money (Chang et al., 2010; Suzuki and Suga, 2010; Rezlescu et al., 2012; Campellone and Kring, 2013; Yu et al., 2014). As an important cognitive function of humans, outcome evaluation is a process in which individuals evaluate results or external feedback caused by their own behaviors (Sun and Luo, 2008). Different feedback leads to distinct emotional experiences. “Gain” or “positive” feedback induces positive emotions, and “loss” or “negative” feedback induces negative emotions (Oosterhof and Todorov, 2008; Todorov et al., 2008b). For outcome evaluation, ERP researchers have focused on feedback-related negativity (FRN), which peaks at frontocentral recording sites between 200 and 350 ms after feedback onset (Holroyd and Coles, 2002).

In daily life, the reality of understanding a person involves integrating information from the observation of their appearances and actual behaviors. For individuals with either concordant or conflicting facial and behavioral trustworthiness, individuals’ reactions and underlying neural substrates warrant further investigation. Previous studies have typically presented faces first followed by feedback. Conversely, less is known about how feedback (outcome evaluation) affects facial trustworthiness judgments. Our study aimed to explore the dissociation between bottom-up and top-down mechanisms in facial trustworthiness processing. Leng et al. (2020) reported a more negative FRN was observed when results were unexpected or negative. Yang et al. (2011) considered that the LPC effect verified the emotion overgeneralization hypothesis of a trustworthy face. Therefore, we hypothesized that trustworthy faces with losses would induce more negative FRN, whereas untrustworthy faces would induce more positive LPC compared with trustworthy faces in the face presentation stage.

## Materials and Methods

### Participants

We calculated the sample size using G Power software (effect size *f* = 0.25, α = 0.05, power = 0.80), and the sample size should be more than 24. A total of 28 college students were recruited *via* advertisements; three participants were excluded due to excessive artifacts in their electroencephalography (EEG) data. Therefore, 25 participants (10 men and 15 women) were included in the final analysis [aged 19–28 years; mean (M) = 23 years, standard deviation (SD) = 3 years]. All participants were right-handed and had normal or corrected-to-normal vision. This study was approved by the institutional research ethics committees of Liaoning Normal University. All participants signed a written informed consent prior to the study.

### Materials

**Figure 1:**
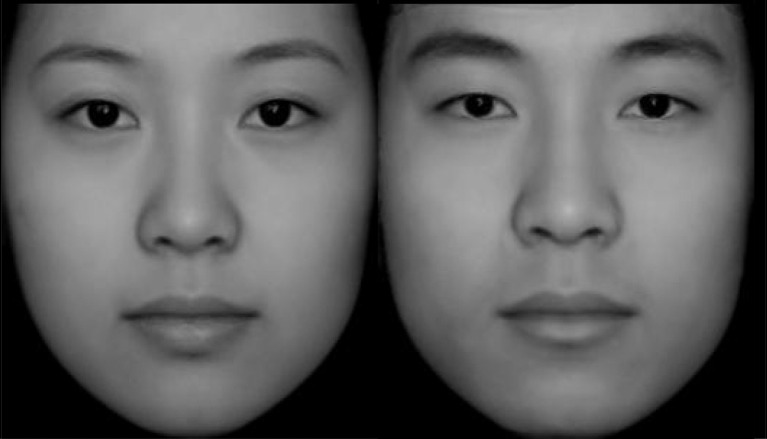
Typical female (left) and male (right) face.

The face images (120 trustworthy faces; 120 untrustworthy faces) used in the experiment were the same as those used by Leng et al. (2020). Neutral emotional faces (109 females, 113 males) were selected from the Chinese facial affective picture system (Gong et al., 2011). The photos were processed using Photoshop and edited to be the same size (260 by 300 pixels). Two psychology postgraduates were requested to rate the trustworthiness of faces and observed that the number of trustworthy faces did not meet the goal of this study. Facial features and structure influence the perception of facial trustworthiness (Todorov et al., 2008a, 2015; Stirrat and Perrett, 2010; Sofer et al., 2015). Typical (average) faces are considered more trustworthy (Sofer et al., 2015; Todorov et al., 2015). We thus combined the original faces with average faces to create new faces to improve the trustworthiness of original faces. The typical female face (Figure 1) was developed by a digital averaging process (PsychoMorph Version 5; Tiddeman et al., 2001) of 109 female faces. The typical female face was then combined with the 109 original male faces, and 109 new female faces (50% typical female face, 50% original female face) were obtained. A similar process was used to transform the 113 male faces (Figure 1). Therefore, we obtained 446 face images including the original 222 images, two typical images averaged from the male and female images, and another 222 images by combining the average face with each of the original images. In total, 10 unclear images were removed from 446 images, and six were selected for the pilot experiment. Finally, 430 valid images were selected. These faces were rated for trustworthiness (from 1 = “very untrustworthy” to 7 = “very trustworthy”) by 33 college students. From the 215 images of male faces, 60 faces that accounted for the top 27% of trustworthy scores were selected as male trustworthy faces; 60 faces that accounted for the bottom 27% of trustworthy scores were selected as male untrustworthy faces. This same process was repeated for the female faces, with 60 trustworthy and 60 untrustworthy faces being finally selected. Finally, a total of 240 faces were selected, comprising 120 trustworthy faces and 120 untrustworthy faces. An independent sample *t*-test revealed a significant difference between the scores for trustworthy and untrustworthy faces (*t*_(238)_ = 31.021, *p* < 0.001, Cohen’s *d* = 4.06). The scores for trustworthy faces (4.87 ± 0.48) were significantly higher than those for untrustworthy faces (2.87 ± 0.51).

### Procedure

**Figure 2:**
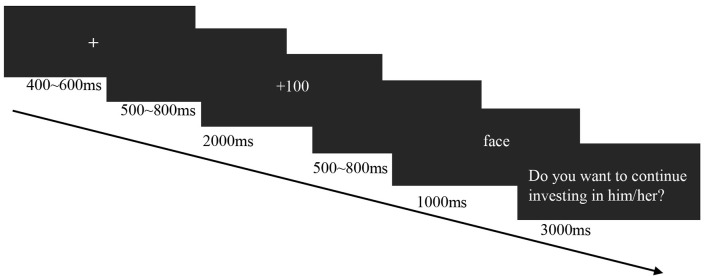
Experimental flow chart.

E-Prime software (Schneider et al., 2002) was used to compile the program and collect participants’ behavioral data. For the experiment, participants were instructed to imagine that they had invested 100 yuan in each person presented in the image. This money would become 400 yuan in the trustee’s hand, and the trustee had two choices: reciprocate 200 yuan to the participant or not. After the participants acknowledged that they understood the instructions, the experimental trial started with presentation of a fixation point in the center of the screen for 400–600 ms. In order to get better baseline correction, a blank screen was subsequently presented for a random duration of 500–800 ms. The feedback of the trustee was then displayed for 2 s “+100,” and “−100” indicated that the participant had earned and lost 100 yuan, respectively. A second blank screen was then presented for a random duration of 500–800 ms. The trustee’s photos were then displayed for 1 s. Finally, the participants were required to decide whether or not to continue investing. The “F” and “J” keys indicated whether to continue or stop investing, respectively (Figure 2). Mapping of keys was counterbalanced between participants. Each image was shown only once in the experiment. The task consisted of 240 trials divided into four blocks. The trial numbers for each combination of outcome signals (gain and loss) and face cues (trustworthy and untrustworthy) were identical.

### Electroencephalography-Event-Related Potential Data Collection and Pre-analysis

An electrode cap (Brain Products GmbH) was used to collect the EEG data. The cap covered 64 scalp sites with tin electrodes arranged according to the 10-20 international placement system. All electrode impedance was maintained below 5 kΩ. EEG signals were sampled at 500 Hz/channel. FCz was used as the reference electrode, and vertical electrooculogram was recorded using an electrode located under the right eye. The bilateral mastoid process was used for re-reference during off-line treatment. The Gratton and Coles ocular correction algorithm of BrainVision Analyzer 2.1 software was used to analyze the EEG data. Channels were marked as artifacts if the signal variation exceeded ±80 μV and were filtered with a low pass of 20 Hz. For analyses of variance (ANOVAs), *p*-values were corrected *via* the Greenhouse–Geisser method, and multiple comparisons were corrected with the Bonferroni method when appropriate. The EEG components of feedback presentation and face presentation stage were analyzed. Epochs were extracted from −200 to 1,000 ms around feedback (face) onset. The data were then baseline corrected according to the 200-ms pre-feedback (pre-face) period. Based on previous studies (Yang et al., 2011; Li et al., 2017; Hu et al., 2018; Leng et al., 2020) and the scalp topographies of each component, we conducted statistical analyses on four ERP components (N1, P200, FRN, and LPC). For the N1 component (amplitude: 80–150 ms), data from Fz, FCz, and Cz electrodes were analyzed. For both the P200 (amplitude: 150–250 ms) and FRN (mean amplitude: 250–400 ms) components, data from Fz, FCz, Cz, CPz, and Pz were analyzed. For the LPC component (mean amplitude: 500–700 ms), data from 15 electrodes (F3/Fz/F4, FC3/FCz/FC4, C3/Cz/C4, CP3/CPz/CP4, and P3/Pz/P4) were analyzed.

## Results

### Behavioral Results

Two-way repeated measures ANOVA was performed with the proportion of participants continuing to invest in four conditions as the dependent variable, [2 (feedback: gain, loss) × 2 (face type: trustworthy face, untrustworthy face)]. Both the main effect (*F*_(1,24)_ = 56.537, *p* < 0.001, $\eta_{p}^{\text{2}}$ = 0.702; *F*_(1,24)_ = 139.747, *p* < 0.001, $\eta_{p}^{\text{2}}$ = 0.853) and the interaction (*F*_(1,24)_ = 14.108, *p* < 0.001, $\eta_{p}^{\text{2}}$ = 0.370) are significant. Simple effects analysis revealed that when the trustee reciprocated, the proportion of participants continuing to invest in trustworthy faces (87.3 ± 12.5%) was significantly higher than that for untrustworthy faces (33.2% ± 22.0%; *t*_(24)_ = 12.537, *p* < 0.001, Cohen’s *d* = 2.506). When the trustee did not reciprocate, the proportion of participants continuing to invest in trustworthy faces (42.6 ± 25.5%) was significantly higher than that for untrustworthy faces (6.6 ± 6.4%; *t*_(24)_ = 7.661, *p* < 0.001, Cohen’s *d* = 1.531).

### Event-Related Potential Results

#### Event-Related Potential Analysis of Feedback

**Figure 3:**
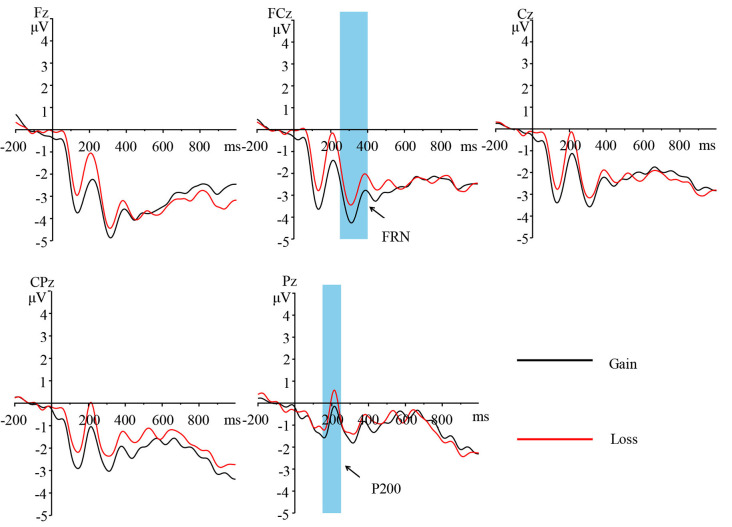
Grand-average event-related potentials (ERPs) of different feedback (gain, loss) at electrode sites Fz, FCz, Cz, CPz, and Pz.

**Figure 4:**
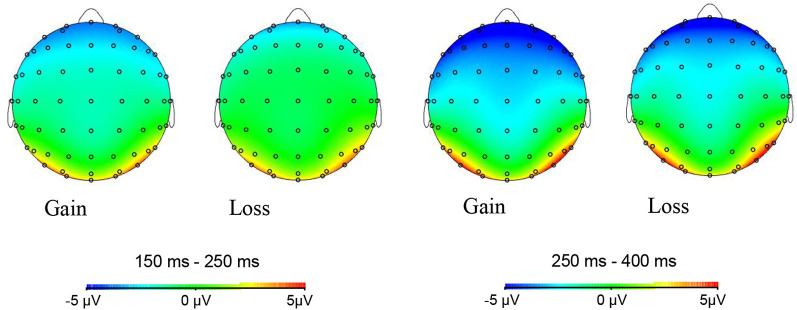
Topographic maps of P200 (left) and feedback-related negativity (FRN; right) in different feedback conditions (gain, loss).

P200 and FRN were induced during the feedback presentation stage. The average trial numbers in the gain and loss conditions were 113 ± 8 and 113 ± 9 (M ± SD), respectively. The number of trials per condition did not differ (*t*_(24)_ = 0.790, *p* = 0.437). The ERPs of different feedback results (gain or loss) at electrode sites Fz, FCz, Cz, CPz, and Pz are shown in Figure 3. Scalp topographies of P200 (150–250 ms) and FRN (250–400 ms) are shown in Figure 4.

##### P200 and FRN

Two-way repeated measures ANOVA was performed with the average amplitude of P200 (150–250 ms) as the dependent variable [2 (feedback: gain, loss) × 5 (electrode: Fz, FCz, Cz, CPz, Pz)]. A significant main effect of feedback was observed, whereby loss (−0.982 ± 2.624 μV) induced a more positive P200 than that of gain (−1.972 ± 2.492 μV; *F*_(1,24)_ = 16.282, *p* < 0.01, $\eta_{p}^{\text{2}}$ = 0.404). A significant main effect of electrode was noted, whereby the electrode located at the Pz site exhibited a higher amplitude at P200 [Pz (−0.577 ± 2.418 μV) > CPz (−1.445 ± 2.642 μV)/Cz (−1.466 ± 2.631 μV)/FCz (−1.598 ± 2.793 μV) > Fz (−2.299 ± 2.850 μV); *F*_(4,96)_ = 7.768, *p* < 0.01, $\eta_{p}^{\text{2}}$ = 0.245]. No significant interaction between feedback and electrode was observed (*F*_(4,96)_ = 1.350, *p* = 0.268).

Similarly, the ANOVA on the FRN amplitude found that the main effects of feedback were marginal significance, whereby loss (−5.166 ± 2.344 μV) induced a more negative FRN than that of gain (−4.570 ± 2.654 μV; *F*_(1,24)_ = 3.15, *p* = 0.089, $\eta_{p}^{\text{2}}$ = 0.116). A significant main effect of electrode was observed, whereby the electrode located at the FCz site exhibited a higher amplitude in FRN [FCz (−5.253 ± 2.693 μV) > Cz (−4.887 ± 2.725 μV); *F*_(4,96)_ = 3.878, *p* = 0.039, $\eta_{p}^{\text{2}}$ = 0.139]. No significant interaction was observed between feedback and electrode (*F*_(4,96)_ = 0.553, *p* = 0.554).

#### Event-Related Potential Analysis of Faces

N1, P200, FRN, and LPC were observed during the face presentation stage. The average trial number of trustworthy faces in both the gain and loss condition was 59 ± 2. The average trial numbers of untrustworthy faces in the gain and loss conditions were 59 ± 1 and 59 ± 2, respectively. No significant differences were observed in the average number of trials among the four cases (*F*_(3,72)_ = 0.958, *p* = 0.415).

**Figure 5:**
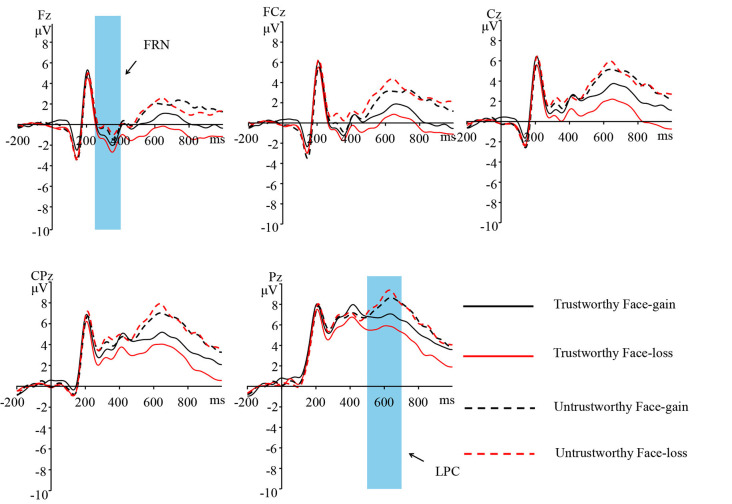
Grand-average ERPs of trustworthy and untrustworthy faces at electrode sites Fz, FCz, Cz, CPz, and Pz in different feedback conditions (gain, loss).

**Figure 6:**
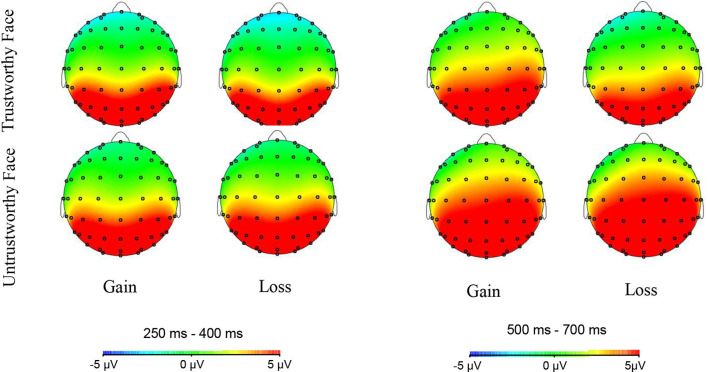
Topographic maps of FRN (left) and late positive component (LPC; right) in different feedback (gain, loss) and facial trustworthiness (trustworthy, untrustworthy) conditions.

ERPs induced by trustworthy and untrustworthy faces at electrode sites Fz, FCz, Cz, CPz, and Pz in different feedback conditions (gain or loss) are shown in Figure 5 Scalp topographies of FRN (250–400 ms) and LPC (500–700 ms) are shown in Figure 6.

##### N1 and P200

A three-way repeated measures ANOVA was performed with N1 amplitude (80–150 ms) as the dependent variable [2 (feedback: gain, loss) × 2 (face type: trustworthy face, untrustworthy face) × 3 (electrode: Fz, FCz, Cz)]. A significant main effect of electrode was noted, whereby the electrode located at the Fz/FCz site exhibited a higher amplitude in N1 [Fz (−5.283 ± 3.030 μV)/FCz (−5.246 ± 3.107 μV) < Cz (−4.516 ± 3.136 μV); *F*_(2,48)_ = 10.662, *p* < 0.001, $\eta_{p}^{\text{2}}$ = 0.308]. A significant interaction between face type and feedback was noted (*F*_(1,24)_ = 5.401, *p* < 0.05, $\eta_{p}^{\text{2}}$ = 0.184). Simple effects analysis revealed that when the feedback was a gain, the N1 amplitude of untrustworthy faces (−5.493 ± 3.042 μV) was significantly more negative than that for trustworthy faces (−4.366 ± 3.331 μV; *t*_(24)_ = 2.978, *p* < 0.01, Cohen’s *d* = 0.595); when the feedback was a loss, the N1 amplitude of trustworthy faces (−5.094 ± 3.254 μV) was not significantly different from that for untrustworthy faces (−5.107 ± 3.438 μV; *t*_(24)_ = 0.031, *p* = 0.976). No other significant main effect or interaction was noted. The ANOVA on the P200 amplitude showed that neither the main effect nor interaction was significant.

##### FRN and LPC

The ANOVA on the FRN amplitude revealed that both the main effect of face type (*F*_(1,24)_ = 18.605, *p* < 0.001, $\eta_{p}^{\text{2}}$ = 0.437) and that of electrode (*F*_(1,24)_ = 85.965, *p* < 0.001, $\eta_{p}^{\text{2}}$ = 0.782) were significant, trustworthy faces (0.946 ± 4.703 μV) induced a more negative FRN than the untrustworthy faces (2.021 ± 5.345 μV), *t*_(24)_ = −4.313, *p* < 0.001, Cohen’s *d* = 0.858, and the electrode located at the Fz/FCz site exhibited a higher amplitude in FRN [Fz (−1.283 ± 4.653 μV)/FCz (−0.816 ± 5.079 μV) < Cz (0.625 ± 5.342 μV) < CPz (3.042 ± 5.519 μV) < Pz (5.849 ± 5.345 μV)].

A significant interaction between face type and feedback was observed (*F*_(1,24)_ = 6.496, *p* < 0.05, $\eta_{p}^{\text{2}}$ = 0.213). No other significant main effect or interactions were noted. Simple effects analysis revealed that when the feedback was a gain, the FRN amplitude of trustworthy faces (1.361 ± 5.186 μV) was not significantly different from that for untrustworthy faces (1.811 ± 5.329 μV; *t*_(24)_ = −1.366, *p* = 0.185); when the feedback was a loss, the FRN amplitude of trustworthy faces (0.531 ± 4.355 μV) was significantly lower than that for untrustworthy faces (2.230 ± 5.479 μV; *t*_(24)_ = −4.614, *p* < 0.01, Cohen’s *d* = 0.920). The FRN amplitude for trustworthy faces with losses (0.531 ± 4.355 μV) was significantly lower than that for trustworthy faces with gains (1.361 ± 5.186 μV; *t*_(24)_ = 2.307, *p* < 0.05, Cohen’s *d* = 0.463). No significant difference between the FRN amplitude for untrustworthy faces with losses (2.230 ± 5.479 μV) and untrustworthy faces with gains (1.811 ± 5.329 μV) was observed (*t*_(24)_ = −1.376, *p* = 0.181).

Similarly, the ANOVA on the LPC amplitude found that both the main effect of face type (*F*_(1,24)_ = 26.048, *p* < 0.001, $\eta_{p}^{\text{2}}$ = 0.520) and that of electrode (*F*_(1,24)_ = 88.961, *p* < 0.001, $\eta_{p}^{\text{2}}$ = 0.788) was significant; untrustworthy faces (4.393 ± 4.729 μV) induced a more positive LPC than the trustworthy faces (2.553 ± 3.832 μV; *t*_(24_ = −5.104, *p* < 0.001, Cohen’s *d* = 1.018), and the electrode located at the Pz site exhibited a higher amplitude in LPC [Pz (7.217 ± 4.412 μV) > CPz (5.314 ± 4.613 μV) > Cz (3.265 ± 4.576 μV) > FCz (1.538 ± 4.343 μV) > Fz (0.031 ± 4.159 μV)].

A significant interaction between face type and feedback was noted, *F*_(1,24)_ = 7.637, *p* < 0.05, $\eta_{p}^{\text{2}}$ = 0.241. No other significant main effect or interactions were noted. Simple effect analysis found that the trustworthy faces with gains (3.138 ± 4.076 μV) induced a more positive LPC than the trustworthy faces with losses (1.967 ± 3.789 μV), *t*_(24)_ = 3.276, *p* < 0.01, Cohen’s *d* = 0.654; there was no significant difference between the LPC amplitude of the untrustworthy faces with gains (4.099 ± 4.616 μV) and the untrustworthy faces with losses (4.688 ± 5.112 μV), *t*_(24)_ = −1.264, *p* = 0.219; there was no significant difference between the LPC amplitude of the untrustworthy faces with gains (4.099 ± 4.616 μV) and the trustworthy faces with gains (3.138 ± 4.076 μV), *t*_(24_ = −1.905, *p* = 0.069; the untrustworthy faces with losses (4.688 ± 5.112 μV) induced more positive LPC than the trustworthy faces with losses (1.967 ± 3.789 μV; *t*_(24)_ = −5.954, *p* < 0.001, Cohen’s *d* = 1.190).

## Discussion

The main findings of our study were that facial trustworthiness and feedback information collectively affected trust behavior. Trustors were more inclined to keep investing in trustworthy trustees or those appearing after positive feedback (gains). This is consistent with previous research, i.e., both initial impressions and previous interactions affect people’s trust in their partners (Chang et al., 2010; Suzuki and Suga, 2010; Rezlescu et al., 2012; Campellone and Kring, 2013; Yu et al., 2014).

At the neural level, we observed that losses induced a more positive P200 than that of gains in the feedback presentation stage. P200 reflects the processing of results, and stimuli with negative valence induce a more positive P200 than do those with neutral valence (Carretié et al., 2001, 2005). Prior research suggests that the P200 is positively correlated with the level of risk taking and reward (Kiat et al., 2016). In no response task or observation task, participants can also induce FRN (Leng and Zhou, 2014). FRN can be considered the main ERP component in outcome evaluation. According to reinforcement learning theory, FRN reflects the difference between expected and actual outcomes, with larger FRN amplitude reflecting larger differences (Gehring and Willoughby, 2002). The affective-motivational hypothesis posits that FRN reflects an evaluation of the affective or motivational significance of errors detected by cognitive monitoring processes. FRN is most pronounced following monetary losses as opposed to monetary gains and does not reflect error detection (Holroyd and Coles, 2002). During the early stage of outcome processing, FRN is more negative for losses than for gains (Hu et al., 2018). Some studies used principal components analysis and thought that the FRN may be an artifact positivity enhanced by rewards (Foti et al., 2011; Proudfit, 2015; Wang et al., 2016). Leng et al. (2020) reported that a more negative FRN was observed when results were unexpected or negative. In our study, the main effects of feedback were marginal significant, which might partly be due to the obscuring of FRN effects by P200 (Rigoni et al., 2010). Loss induced a more negative FRN than that of gain, which was consistent with the FRN literature (Gehring and Willoughby, 2002; Yu et al., 2007; Li et al., 2010; Foti et al., 2011).

During the face presentation stage, untrustworthy faces with gains induced a more negative N1 amplitude than that of trustworthy faces with gains. Indeed, negative stimuli induce a more negative N1 amplitude than do positive stimuli (Carretié et al., 2004; Smith et al., 2005; Sun et al., 2012), and early processing bias of threat information biases attention toward threat information (Öhman and Mineka, 2001; Olofsson et al., 2008). This reflects an evolutionary adaptative mechanism to rapidly deal with threats (Carretié et al., 2004, 2006). Untrustworthy faces are more threatening than trustworthy faces, thus inducing more negative N1. The lack of a significant difference in N1 between untrustworthy faces with losses and trustworthy faces with losses could be because participants had already experienced the threat when the feedback was a loss, i.e., they tended to think the subsequent face was untrustworthy and paid less attention to it.

FRN can appear after feedback presentation and after cue stimuli before feedback presentation (Osinsky et al., 2014; Li et al., 2017). In the ultimatum game, unfair results and seeing the face of the unfair proposer before the results are presented will induce FRN. We found that trustworthy faces induced a more negative FRN than the untrustworthy faces, which is consistent with previous research (Chen et al., 2012; Li et al., 2017). Attractive faces are perceived as more trustworthy (Oosterhof and Todorov, 2008; Xu et al., 2012; Lee et al., 2017). Chen et al. (2012) found that attractive faces induced a more negative FRN than unattractive faces; attractive trustees’ betrayal are unexpected for trustors. Li et al. (2017) used multiround trust games to assess how individuals distinguish trustworthiness of others and observed that during the later stages of the game, untrustworthy partners induced larger FN amplitude than did the trustworthy partners.

When feedback is a loss, individuals are more inclined to expect that the subsequent face presented is untrustworthy. When a trustworthy face appears, this violates expectations and elicits greater conflict. Therefore, trustworthy faces induce a more negative FRN than do untrustworthy faces. When feedback is a gain, individuals are more inclined to expect that the subsequent face presented is also trustworthy. Hence, trustworthy faces with losses induce greater negative FRN than do trustworthy faces with gains. If results are inconsistent with one’s expectations, a more negative FRN will be induced. In this regard, a negative result may not necessarily induce a more negative FRN. Expectations for trustworthy faces are generally greater, and the discrepancy between trustworthy faces and associated behavior leads to greater conflicts, thus inducing a more negative FRN. FRN is positively correlated with the level of risk taking and reward (Kiat et al., 2016). Wang et al. (2019) reported that medial frontal negativity (MFN) reflected both probability weight and money magnitude processes, and low-probability options or small magnitude induced a more pronounced MFN. Untrustworthy faces and losses indicate low-probability options or small magnitude. We observed that when the feedback was a gain, the FRN amplitude of trustworthy faces was not significantly different from that of untrustworthy faces. Further, no significant difference was observed between the FRN amplitude of untrustworthy faces with losses and untrustworthy faces with gains. In sum, the effects of conflict on FRN were greater than the effects of low-probability options or small magnitude.

Compared to trustworthy faces, untrustworthy faces induced a more positive LPC, which is consistent with previous research (Yang et al., 2011; Lischke et al., 2018). Yang et al. (2011) considered that the LPC effect was consistent with the prediction of the emotion overgeneralization hypothesis of a trustworthy face. Untrustworthy faces can increase the activity of amygdala, which plays an important role in motivation evaluation (Todorov et al., 2008b). LPC is enhanced by increased motivated attention (Schupp et al., 2000, 2004a,b; Langeslag et al., 2007; Yang et al., 2011) implying that more attention is allocated to untrustworthy faces than to trustworthy faces. We observed an interaction between face type and feedback; trustworthy faces with gains induced a more positive LPC and generated more motivated attention than did trustworthy faces with losses. When feedback was a gain, participants were more inclined to expect that the subsequent face presented would be trustworthy; hence, they paid more attention to trustworthy faces. Conversely, when feedback was a loss, participants were more inclined to expect that the subsequent face to be presented would be untrustworthy; hence, they paid more attention to untrustworthy faces. In sum, trustworthy faces with gains generated more motivated attention in participants than did trustworthy faces with losses.

Bottom–up and top–down processes are integrated for facial trustworthiness perception at different stages. During the face presentation stage, untrustworthy faces with gains induced a more negative N1 compared to that for trustworthy faces with gains, reflecting early perception of threat information. In the loss condition, no differences in N1 amplitude were noted for facial trustworthiness, suggesting that top–down processing (feedback) exerted a greater impact during the early stage. Trustworthy faces with losses induced a more negative FRN compared to that for trustworthy faces with gains and untrustworthy faces with losses. Outcomes that did not meet expectations induced more negative FRN, suggesting that top–down and bottom–up processing are integrated in the medium term. Untrustworthy faces induced more positive LPC compared to that for trustworthy faces and generated more motivated attention; trustworthy faces with gains induced more positive LPC than did trustworthy faces with losses and generated more motivated attention. Regardless of previous feedback, no significant difference was noted in the amplitude of LPC induced by untrustworthy faces, implying that bottom–up processing (facial trustworthiness) exerted a greater impact during the later stage.

With regard to face stimuli, a degree of synthesis was employed even though some original natural faces were utilized. Future experiments should construct larger face stimulus libraries to generate more ecologically valid experimental materials. In our study, participants were instructed to imagine that they had invested 100 yuan. Future studies should also consider actual investment in individuals and present the faces of investors after a period of time. For the ERP analysis, we would try to use principal components analysis to better compare with other studies and increase the sample size to verify the stability of the results.

## Data Availability Statement

The raw data supporting the conclusions of this article will be made available by the authors, without undue reservation, to any qualified researcher.

## Ethics Statement

The studies involving human participants were reviewed and approved by institutional research ethics committees of Liaoning Normal University. The patients/participants provided their written informed consent to participate in this study.

## Author Contributions

ZJ conceived this study. HL participated in performing the study and writing the manuscript. YL, QL, QW, and DL participated in editing the manuscript. All authors contributed to the article and approved the submitted version.

## Conflict of Interest

The authors declare that the research was conducted in the absence of any commercial or financial relationships that could be construed as a potential conflict of interest.
